# Modified gingivoplasty for hereditary gingival fibromatosis: two case reports

**DOI:** 10.1186/s12903-022-02411-y

**Published:** 2022-11-23

**Authors:** Xin Huang, Wenjun Zhu, Xinfang Zhang, Yun Fu

**Affiliations:** 1 Hospital of Stomatology, Guanghua School of Stomatology, Sun Yat-Sen University, 56 Ling-yuan West Road, Guangzhou, 510055 Guangdong China; 2 Guangdong Provincial Key Laboratory of Stomatology, Sun Yat-Sen University, Guangzhou, Guangdong China

**Keywords:** Hereditary gingival fibromatosis (HGF), Keratinized gingiva, Modified gingivoplasty, Case report

## Abstract

**Background:**

Hereditary gingival fibromatosis (HGF) is characterized by sub-epithelial fibromatosis of keratinized gingiva resulting in a fibrotic enlargement of keratinized gingiva. The treatment choice is gingivectomy, which can be performed with an internal or external bevel incision conventionally. However, both techniques can hardly resume the natural status of gingiva, and have a certain recurrence rate, especially in the cases which have limited width of attached gingiva.

**Case description:**

Two cases of HGF with the chief complaint of difficulty in mastication, pronunciation, and poor esthetics were presented. After the initial periodontal therapy, a novel gingivoplasty modified with a crevicular incision was applied. A full thickness flap above the mucogingival junction and a split flap below the junction were raised. Then, fibrotic connective tissue was completely eliminated and keratinized gingival epithelium was preserved. The fibrotic alveolar bone was shaped by handpiece and bur. Finally, the flap was apically repositioned and sutured. Twelve months after surgery, the gingiva recovered with normal color, contour and consistency.

**Conclusions:**

Compared to traditional gingivectomy, modified gingivoplasty which focuses on eliminating pathological fibrotic connective tissue can completely resume the natural appearance of gingiva and demonstrate no tendency of recurrence.

## Background

Hereditary gingival fibromatosis (HGF) is a rare disorder characterized by a benign, slowly progressive, fibrous gingival overgrowth that can appear as an isolated disorder (non-syndromic) or as part of a syndrome (syndromic) and it may be localized or generalized [1]. Non-syndromic form is the most common genetic form of gingival fibromatosis with both autosomal dominant and recessive modes of inheritance. The prevalence of HGF is low (1:175,000 according to phenotype and 1:350,000 according to genotype) [2] and the candidate gene varies among different races. Linkage analysis of families suffering from a revealed several regions on chromosomes that may potentially contain pathogenic variants of genes (chromosomes 2p21-p22 in a Brazilian family; chromosomes 5q13-q22, 2p22.3-p23.3 and 11p15 in several Chinese families) contributing to this condition [3–6].

According to the classification of periodontal disease by world workshop in 2017, HGF is classified as non-plaque-induced gingival disease [7].The diagnosis of HGF is mainly based on a thorough clinical evaluation and histopathology analysis of gingiva biopsy. Gingival overgrowth is the single most common manifestation in non-syndromic forms, whereas extraoral symptoms such as generalized hypertrichosis, mental retardation, epilepsy, facial dysmorphia, nails dysplasia, and organs malformations are characterized in syndromic forms [1]. The overgrowth tissue may cover the crowns of the teeth and cause periodontitis, occlusion disorder, delayed tooth eruption and facial disfigurement [8, 9]. In the late stage, the fibrous overgrowth implicates to the whole gingival tissue, leading to functional impairment, damage of appearance and eventually teeth loss. The pathological characteristics of gingival fibromatosis are more sub-epithelial fibroblast proliferation, greater collagen and fibronectin synthesis and reduction in the matrix metalloproteinases (MMPs) related collagen degradation [10]. The major contributing component of fibrotic gingiva is the excessive production of the structural protein collagen type I (Col I) [11]. Most of them exist in the inner layer of the connective tissue. And the histopathology characteristic may show as acanthosis, long extended rete pegs, increased collagen bundles, distinctive directions of collagen bundles, few blood vessels, increased amount of elastic and oxytalan fibers, osseous calcifications, and so on [12].

**Fig. 1:**
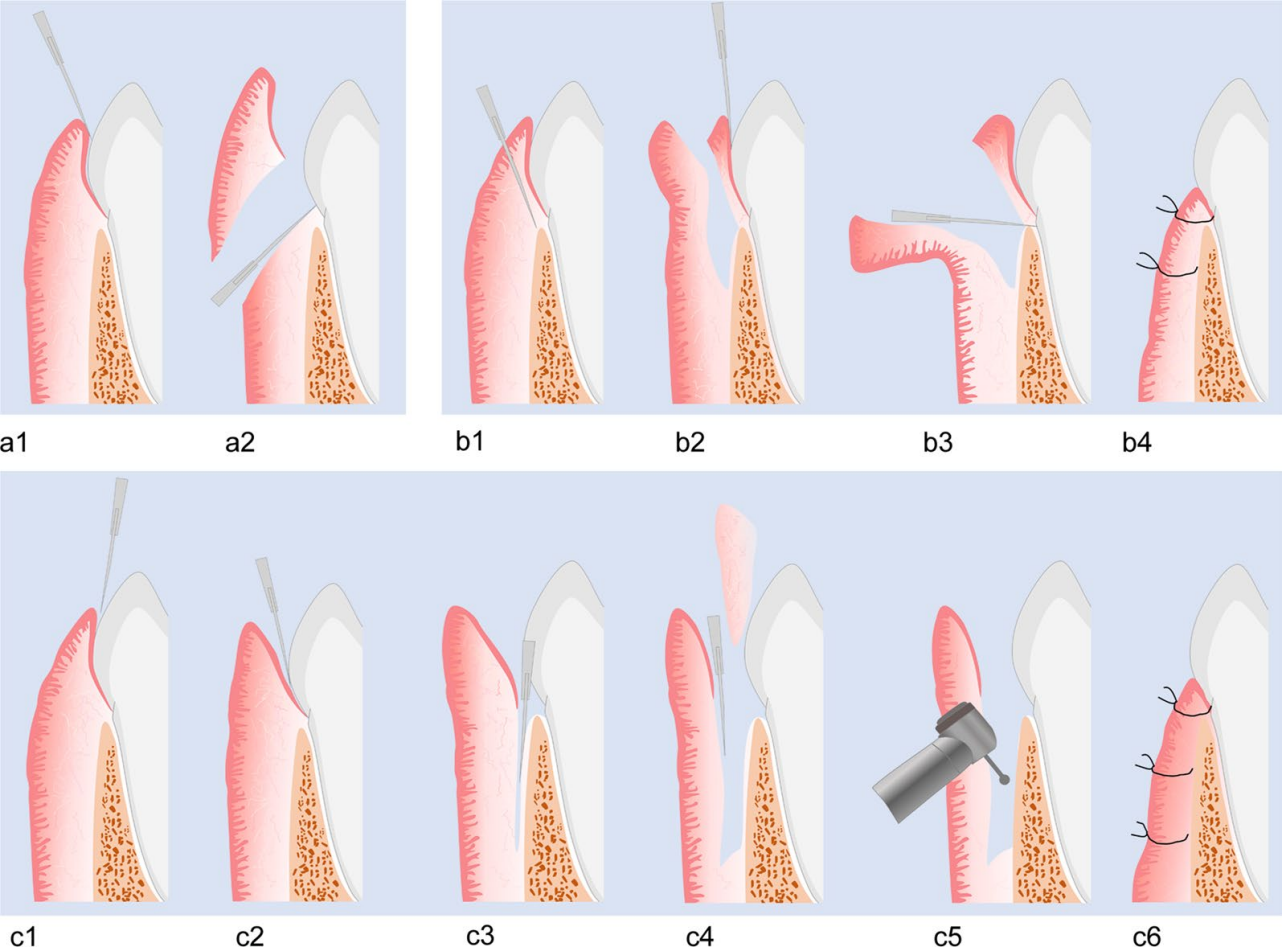
The schematic illustration of traditional and modified surgery for HGF. One of the traditional operations **a1–a2** removes the overgrown tissue mainly by the external bevel incision. Another traditional operation consists with an internal bevel incision **b1**, crevicular incision **b2** and interdental incision **b3**, and the flap is apically repositioned **b4** Both of them have a limit on the complete removal of overgrown tissue and the preservation of gingival epithelial layer. The first incision of modified gingivectomy enters along to the sulcus **c1** and down to the alveolus **c2** Full thickness flap is made superior to the mucogingival junction and split thickness flap is raised inferior to the mucogingival junction **c3** Dependent upon its colour and texture, the fibrotic connective tissue is removed **c4** The use of handpiece and bur is for the osteoplasty **c5** Flap edges are then approximated to the cemento-enamel junction (CEJ) with the flap apically repositioned **c6**

HGF does not resolve spontaneously and various modalities can be administered for this including conventional external or internal bevel gingivectomy procedure, electrocautery, or diode laser [13, 14]. The external bevel technique is comparatively simple and time saving (Fig. 1 a1–a2) and the internal bevel gingivectomy makes it possible to view the bone crest and its relationship with the cemento-enamel junction (CEJ) (Figs. 1 b1–b4, 2). However, both techniques can hardly achieve a satisfactory result (eg. lead to a nodular appearance, sustained redness/swollen) for the resection of keratinized gingival tissue, especially in the cases which have limited width of attached gingiva [15, 16]. On the other hand, certain recurrence rate occurs. According to researches, the overall recurrence rate of HGF is 33.85% within a median time period of 12 months after traditional gingivectomy [17–19] and it may be related to the age, genetics, surgical technique, location of hyperplasia and so on [20]. It has also been demonstrated that recurrence is faster in areas with dental plaque accumulation [21]. Laser is preferred due to the increased comfort of patient, decreased postoperative healing time and decreased volume of hemorrhage. However, lateral heat damage, lack of control over penetration of laser, required technical skill of the operator, and higher cost limit its application [13].

**Fig. 2:**
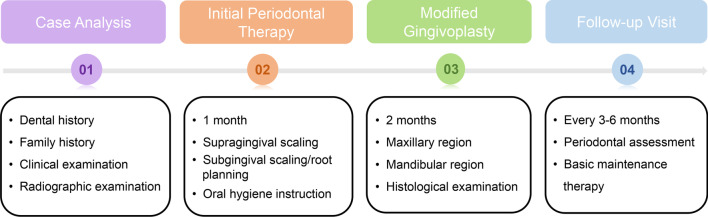
Schematic overview of the approach used in the 2 cases

Hence, we introduced a modified gingivoplasty to improve surgical techniques which focus on eliminating fibrotic connective tissue and preserve keratinized gingival epithelial layer during the operation (Fig. 2). 12 months after surgery, the color, contour and consistency of the gingiva became normal with no signs of inflammation (Figs. 5 and 7).

### Case presentations

#### Case 1

A 20-year-old woman was referred to the periodontics department, Hospital of Stomatology for the management of her periodontal enlargement. The patient's chief complaint was painless swollen gums, difficulty in mastication, phonetics, and unpleasing appearance. Her medical history was non-contributory and she was taking no therapeutic drug regularly. In the course of taking family history, she reported another case of gingival enlargement in her biological sister.

**Fig. 3:**
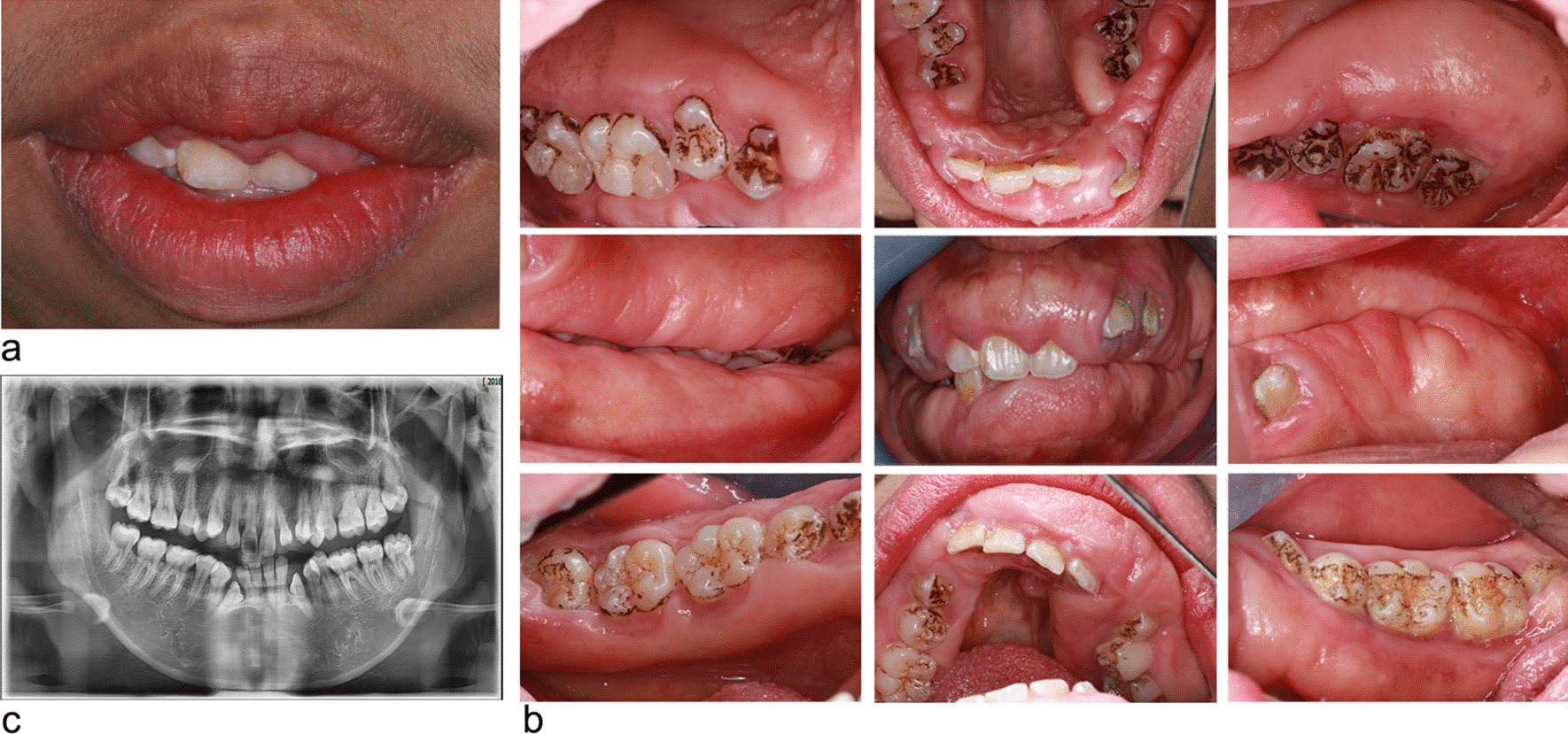
The initial appointment of Case 1. **a** Preoperative extraoral aspects. **b** The intraoral examination (right, central and left maxillary palatal views; right, central and left occlusal views; right, central and left mandibular lingual views). **c** Panoramic radiograph

Physical examination allowed to exclude major dysmorphic features of the face, hands, and nails as well as hypertrichosis. No deafness or intellectual disability was evidenced. On extra-oral examination, the patient presented with an open lip posture and convex profile (Fig. 3a). The intraoral examination revealed a calculus index (CI) [22] of 1–2, and plaque index of 1–2. The enlarged gingiva was diffused, pink in color and dense in consistency covering most part of teeth on buccal and lingual/palatal aspects in both maxillary and mandibular arches. There were diastemas between the teeth and over-bite was also present (Fig. 3b). The maxillary and mandibular anterior teeth showed migration with diastemas. Few carious teeth were noted. Panoramic radiograph revealed no significant changes in alveolar bone (Fig. 3c).

**Fig. 4:**
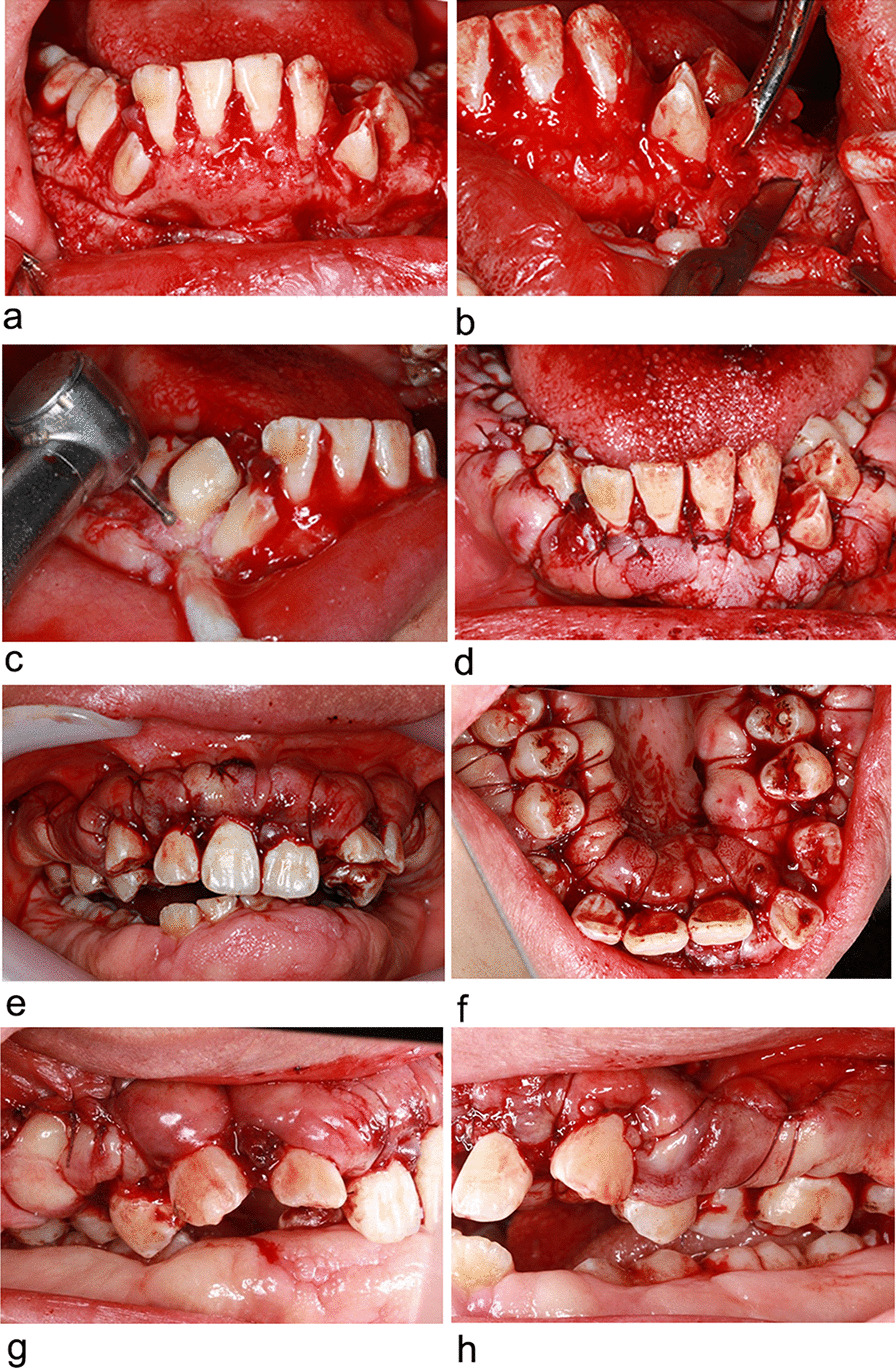
Case 1. The clinical views of modified gingivoplasty. For the mandible, the full thickness flap was raised with the crevicular incision **a** The fibrotic connective tissue was entirely removed according to its colour and texture **b** The osteoplasty was applied to remove the fibrotic alveolar bone **c** After the debridement, the flap was repositioned apically and sutured **d** For the maxilla, Intraoral views(central, right and left occlusal views of maxilla) **e, g, h**; Central maxillary palatal views **f**

On the basis of these findings, a diagnosis of non-syndromic hereditary gingival fibromatosis (HGF) was given. With the consent of the patient and her family, non-surgical periodontal treatment which comprised sub-gingival scaling and root planning and oral hygiene instruction was initially taken. Next, a bilateral posterior superior alveolar nerve block was administered with a supplemental local anesthesia obtained through labial and palatal infiltration. After strict disinfection, gingivoplasty surgery in both labial/buccal and palatal/lingual sides in the maxilla was performed. Crevicular incision was made along to the sulcus the using a cconventional scalpel (11/12/15c) and down to the alveolus and then, full thickness flap was raised above the mucogingival junction (Fig. 4a). The fibrotic connective tissue and alveolar bone were exposed. It’s not difficult to distinguish the pathological tissue from the outside soft tissue, for the pathological part is very hard in texture and pale in colour. Therefore, depending on the texture and color, the fibrotic connective tissue was resected and the gingiva was scalloped in shape, with slightly thinner margins (Fig. 4b). The handpiece and bur were used to remove the outside fibrotic alveolar bone and reshape normal physiological alveolar anatomy (Fig. 4c). The split thickness gingival flap was raised below the mucogingival junction. After debridement, the flap was repositioned apically and sutured with absorbable suture (polyglycolic acid braid with short term resorption) (Fig. 4d). With a bilateral inferior alveolar nerve block anesthesia and labial and palatal infiltration anesthesia, the operation in the mandible was utilized 4 weeks after the one in the maxilla (Fig. 4e–h). The patient was advised to continue taking the antibiotic (amoxicillin, 500 mg tds) and fluid intake after operation for 3 days. Intense exercise and brushing teeth within 24 h after operation should be avoided. A 0.12% chlorhexidine gluconate rinse was prescribed for administration twice a day for one week. The patient could eat without pain 7 days after the operation. Comfortably eating was achieved 14 days after the suture were removed. Complete healing occurred 3 months after the operation.

**Fig. 5:**
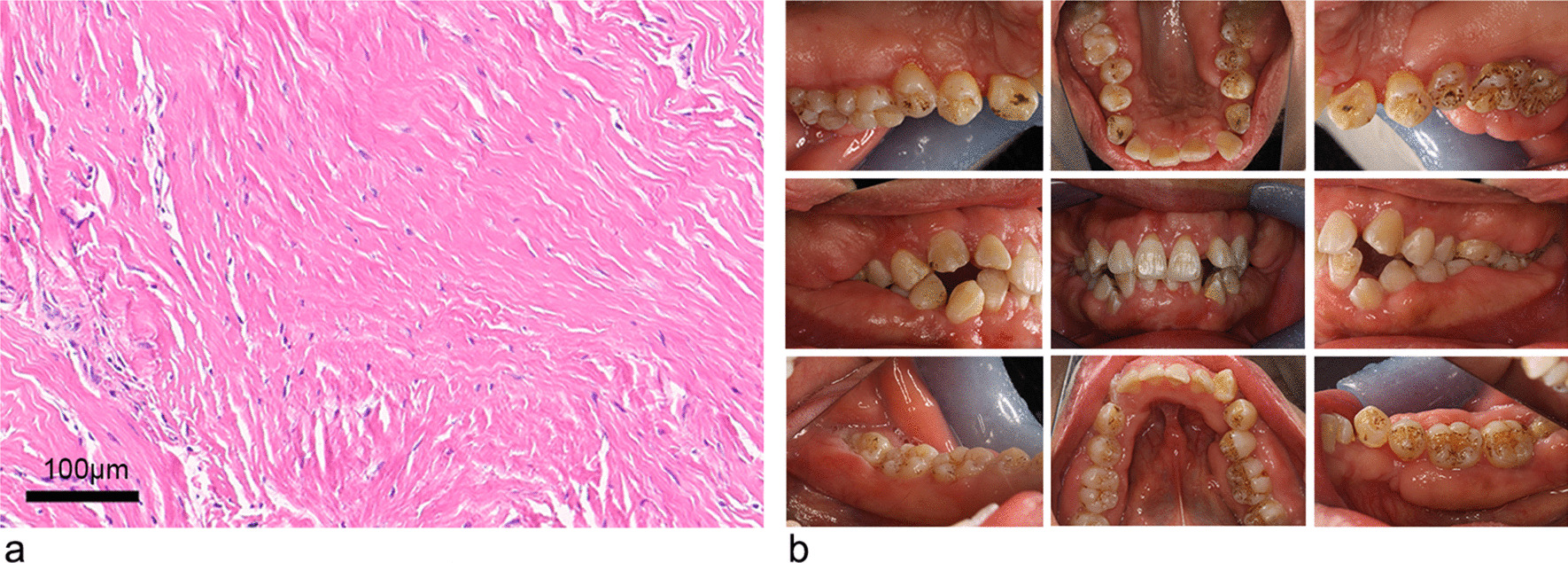
Case 1. **a** Histopathological examination of the lesion. **b** Intraoral views(right, central and left maxillary palatal views; right, central and left occlusal views; right, central and left mandibular lingual views) at 12 months after the last modified gingivoplasty

Postoperatively, the removed gingival tissue was examined pathologically (Fig. 5a). After haematoxylin and eosin (H&E) staining, histopathology analysis of the specimen showed that mature collagenous connective tissue with collagen bundles arranged in a parallel manner. Connective tissue was relatively avascular along with scanty inflammatory cell infiltration, mainly lymphocytes and plasma cells infiltration. These finding corresponded with the characteristics of HGF.

12 months after the first surgery (Fig. 5b), no pain or discomfort was complained and no inflammation was found throughout the observation period. The crowns adequately exposed without any tendency of recurrence. The oral hygiene was reinforced. After the surgery, the patient underwent a regular periodontal assessment and basic maintenance therapy every 3 to 6 months and lost after one year follow up. Imperfectly, the patient gave up the orthodontic treatment for the financial problem though it was strongly advised.

#### Case 2

**Fig. 6:**
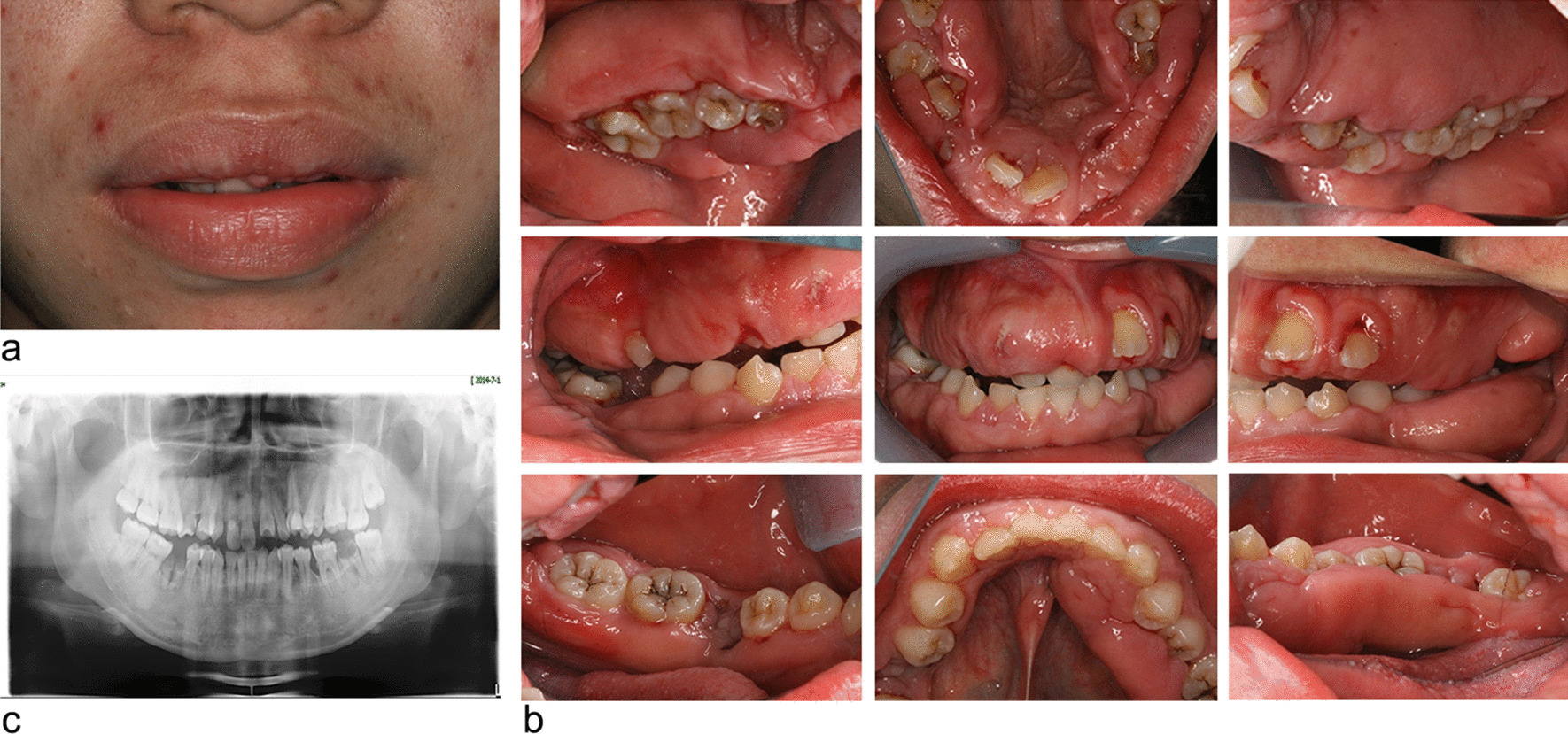
The initial appointment of Case 2. **a** Preoperative extraoral manifestation. **b** The intraoral views (right, central and left maxillary palatal views; right, central and left occlusal views; right, central and left mandibular lingual views). **c** The pantomogram

The patient was a 22-year-old female when she first visited the periodontics department, Hospital of Stomatology with a chief complaint of progressive gingival enlargement to cover the entire permanent dentition. In the past 2 years, the enlargement led to difficulty in mastication, pronunciation, and poor esthetics. She had not reported gingival pain and spontaneous bleeding. Her father also had a history of similar gingival enlargement. There was no history of taking medicine, systemic disorder or drug allergy. No signs of mental disorder, hormonal changes, or deformities of nail development were observed. Extraoral examination showed an open-lip posture (Fig. 6a). Intraoral examination revealed poor oral hygiene, with a large amount of debris at the gingival margin. Both the free and attached gingiva showed hyperplasia and hypertrophy in the maxillary and mandibular region. In the severe area, the pathological gingival tissue which had a firm and fibrous consistency covers three-quarters to the entire of the crowns. No noticeable mobility of teeth was detected. The displacement of anterior teeth and premolars could be seen and the dental caries in posterior teeth as well the residual roots of tooth #37&#46 were detected (Fig. 6b). The panoramic radiograph showed no obvious horizontal and vertical bone loss. Carious teeth in the posterior region and residual roots of tooth #37&#46 were noted (Fig. 6c).

The patient was diagnosed with non-syndromic HGF, multiple dental caries and residual root of tooth #37&#46. With the consent of the patient and her family, sub-gingival scaling/root planning and oral hygiene education were performed in the first month with no significant improvement in the gingival enlargement. Surgical treatment was performed in the maxillary and mandibular region in sequence using a modified gingivoplasty combined with a crevicular incision and apically positioned flap as mentioned. During surgery, the fibrotic connective tissue was removed to the maximum, and the keratinized gingiva was retained. Besides, the residual roots were extracted. The patient was given the same postoperative guidelines as in case 1.

**Fig. 7:**
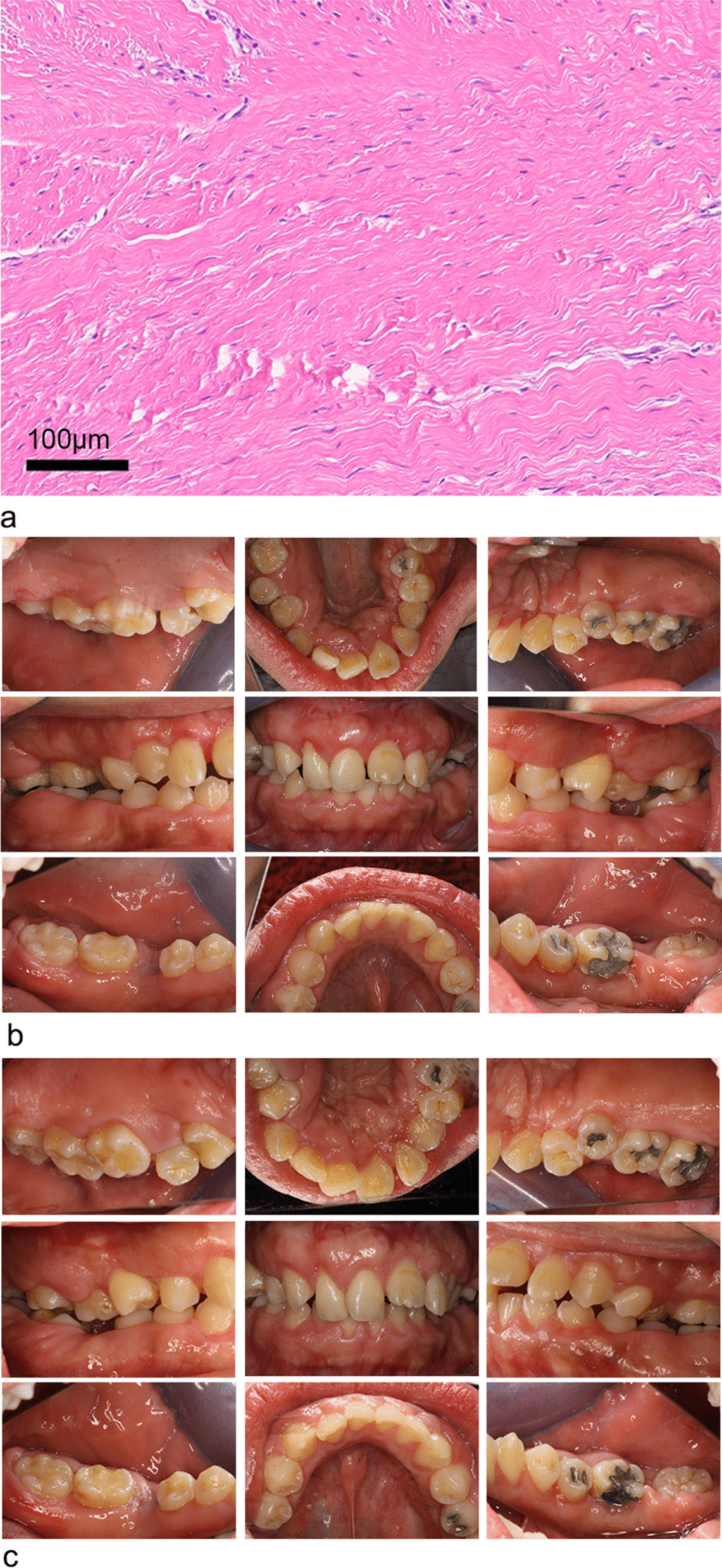
Case 2. **a** Histopathological examination of the lesion. Intraoral views(right, central and left maxillary palatal views; right, central and left occlusal views; right, central and left mandibular lingual views) at 3 months **b** and 12 months **c** after the last modified gingivoplasty

The removed hyperplastic gingival tissues were measure by a routine pathological examination. The connective tissue was presented as fibrous and avascular, and has densely-arranged collagen-fiber bundles arranging in all directions, numerous fibroblasts, and mild chronic inflammatory cells infiltration (Fig. 7a).

Three months after the gingivoplasty, the patient was able to maintain good oral hygiene, and the crowns were adequately exposed. Moreover, the gingival morphology in the upper and lower dentition significantly was improved (Fig. 7b). During the period, the restoration for caries and occlusal therapy had been finished. Twelve months after the treatment, the patient still maintained good oral hygiene without periodontal inflammation (Fig. 7c). There was no significant evidence on the recurrence of gingival hypertrophy. It is important to conduct basic periodontal therapy, plaque and calculus control, and oral hygiene maintenance either before, during, or after surgery. Hence, the patient kept undergoing a regular periodontal assessment and basic maintenance therapy every 3 to 6 months and lost after one year follow up. The patient also gave up the orthodontic treatment for the financial problem.

## Discussion and conclusions

As a hereditary disease, the etiology of HGF is not yet clear, and not any effective drug has been developed. Therefore, the existing effective treatment methods can only be gingivectomy. How to achieve better surgical results and reduce postoperative recurrence rate becomes very important.

The gingivectomy of HGF can be performed with an internal or external bevel incision traditionally [7, 8] and the latter is the principle method adopted. The external bevel technique is comparatively simple and time saving, allowing gingivectomy to be performed without raising a flap and avoiding the need for suturing. However, the hypertrophic tissues resected through this technique are mainly keratinized epithelium and the upper layer of gingival connective tissue which are not the contributing factor of the enlargement (Fig. 1 a1–a2).

On the one hand, the major contributing component of fibrotic gingiva is the excessive production of the structural protein collagen type I (Col I) [14]. Most of them exist in the inner layer of the connective tissue as well as the surface of alveolar bone. In the external bevel technique, the pathological collagenous connective tissue with collagen bundles in the inner layer of lamina propria and close to alveolar bone were retained. This may explain the undesirable clinical effect of the method. The exposure of the fibrotic tissue without covering with keratinized epithelium may lead to a nodular appearance of gingiva and a relatively high recurrence rate [9, 10]. On the other hand, it should be noted that the host's innate and acquired immune systems are important defense mechanisms that protect periodontal tissues from attacking and invasion of periodontal pathogens, thus preventing infection. As the first mechanical barrier of periodontium against exogenous infection and a part of the innate immune response to bacterial invasion, keratinized epithelium plays a key role in the health of periodontal tissue [23]. The decrease in the number of keratinized epithelium may weaken the antimicrobial ability of gingiva, resulting in the invasion of periodontal pathogen [24]. The sustained redness or swollen after surgery may thus happens.

Internal bevel gingivectomy makes it possible to view the bone crest and its relationship with the cemento-enamel junction (CEJ). Nevertheless, the fibrotic connective tissues may just be partially removed and the keratinized epithelium is still excised to some extent in the surgery, resulting a compromised effect afterwards (Fig. 1 b1–b4). According to the observations reported by investigators, the remaining of the pathological fibrotic tissue may have an influence on gingival healing and the recurrence of overgrowth [13]. It should be noted that pathological fibrotic connective tissue in the inner layer of lamina propria is the main contributing factor of gingival enlargement. In line with the principle of cause-oriented treatment, the best therapeutic result can only be achieved when pathological factors in the lesion have been entirely eliminated while the protective tissue and structures related to immune response can be saved as much as possible.

Therefore, in order to maximize the removal of fibrotic tissue and saving keratinized epithelium, a modified gingivoplasty was introduced. Instead of cutting off comparatively normal tissue, in our method, pathological part of the lesion was just excised. Since the first mechanical barrier against bacterial invasion and the natural structures of periodontium were almost retained, the gingiva may recover health with no redness or swollen and other sign of inflammation. At the same time, as pathological part of gingiva including fibrotic connective tissue and alveolar bone were removed to the most, the natural appearance may be completely restored and demonstrate no tendency of recurrence 12 months after the surgery.

However, there are several challenges faced during performing the surgery. In the HGF cases, the gingival enlargement occurred in both labial/buccal and palatal/lingual sides in the maxilla and mandible, and almost covered the entire crown. Therefore, in order to limit the operation time duration and decrease the post-operative reaction, the gingivoplasty surgery in the maxilla and mandible was performed separately. The split thickness gingival flap was raised below the mucogingival junction which is another challenge faced during performing the technique. Sufficient tension reduction of the gingival flap is the key for the success of the surgery. Therefore, the fibrotic connective tissue should be removed to the most. After sufficient debridement, the flap was able to be repositioned apically and sutured perfectly.

In our study, compared to traditional gingivectomy, modified gingivoplasty which focus on eliminating fibrotic connective tissue can completely resume the natural appearance of gingiva and demonstrate no tendency of recurrence.

## Data Availability

All data and material supporting our conclusions are contained within the manuscript.

## Abbreviations

HGF: Hereditary gingival fibromatosis
MMPs: Matrix metalloproteinases
CI: Calculus index
H&E: Haematoxylin and eosin
Col I: Collagen type I
CEJ: Cemento-enamel junction
